# Case Report: Multidisciplinary Approach for a Rare Case of Thymic Vascular Malformation

**DOI:** 10.3389/fsurg.2020.624615

**Published:** 2021-01-12

**Authors:** Federico Raveglia, Laura Moneghini, Maurizio Cariati, Alessandro Baisi, Angelo Guttadauro, Ugo Cioffi, Marco Scarci

**Affiliations:** ^1^Department of Thoracic Surgery, Aziende Socio Sanitarie Territoriali-Monza, Monza, Italy; ^2^Department of Pathology, Aziende Socio Sanitarie Territoriali Santi Paolo e Carlo, Milano, Italy; ^3^Department of Radiology, Aziende Socio Sanitarie Territoriali Santi Paolo e Carlo, Milano, Italy; ^4^Department of Thoracic Surgery, Aziende Socio Sanitarie Territoriali Santi Paolo e Carlo, Milano, Italy; ^5^Istituto Clinico Zucchi, University of Milan, Milano, Italy

**Keywords:** case report, residual thymus, venous vascular malformation, vessel embolization, multidisciplinary approach

## Abstract

We report the rare case of a 2.5 cm in size mass diagnostic for residual thymus associated with venous vascular malformation (ISSVA classification, 2008) in a 58 years old man. Diagnosis was obtained only after surgical removal that was complicated by a sudden massive bleeding (about 1,500 cc) requiring emergency conversion to median sternotomy. Difficulty in preoperative diagnosis, rarity of histologic pattern, and surgical challenges make this case very interesting for surgeons, pathologists and radiologist. Our message, dealing with mediastinal masses, is: (a) differential diagnosis between the more frequent solid antero-superior mediastinal tumors and vascular malformation should be always considered (b) preoperative angiography should always be performed in case of uncertain diagnosis (c) coil embolization should always be considered to reduce potentially fatal bleeding (d) histologic differentiation with other thymic neoplasms must be always considered.

## Introduction

Mediastinum is a space where several vascular or solid pathologic masses can arise. Vascular ones are rare and, according to first classification by Mulliken and Glowacki (1), have been distinguished in tumors (hemangioma) and malformations, as then confirmed by the International Society for the Study of Vascular Anomalies (ISSVA) (2, 3). Malformations and tumors are hardly discernible at routine clinical staging, as well as others solid mediastinal neoplastic disease (4).

## Case Description

**Figure 1:**
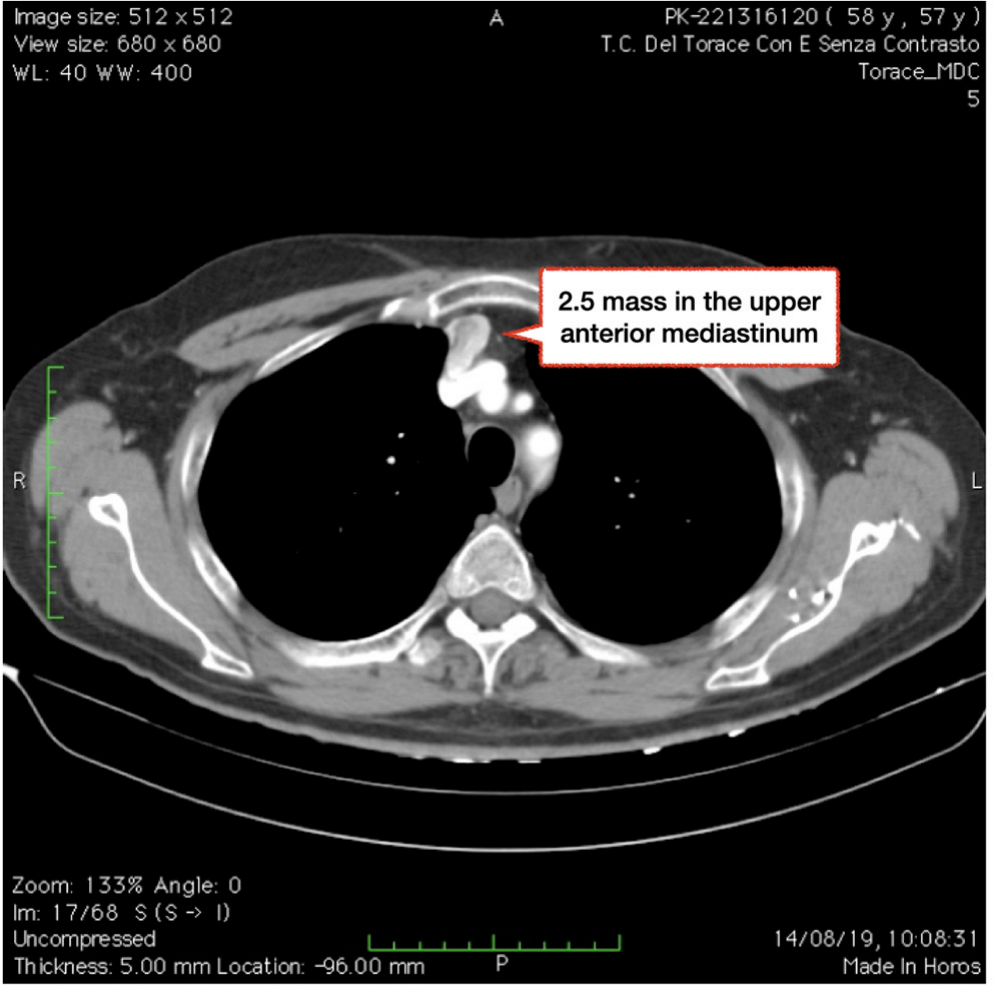
CT scan showing a 2.5 cm mass in the upper anterior mediastinum, with positive contrast enhancement.

**Figure 2:**
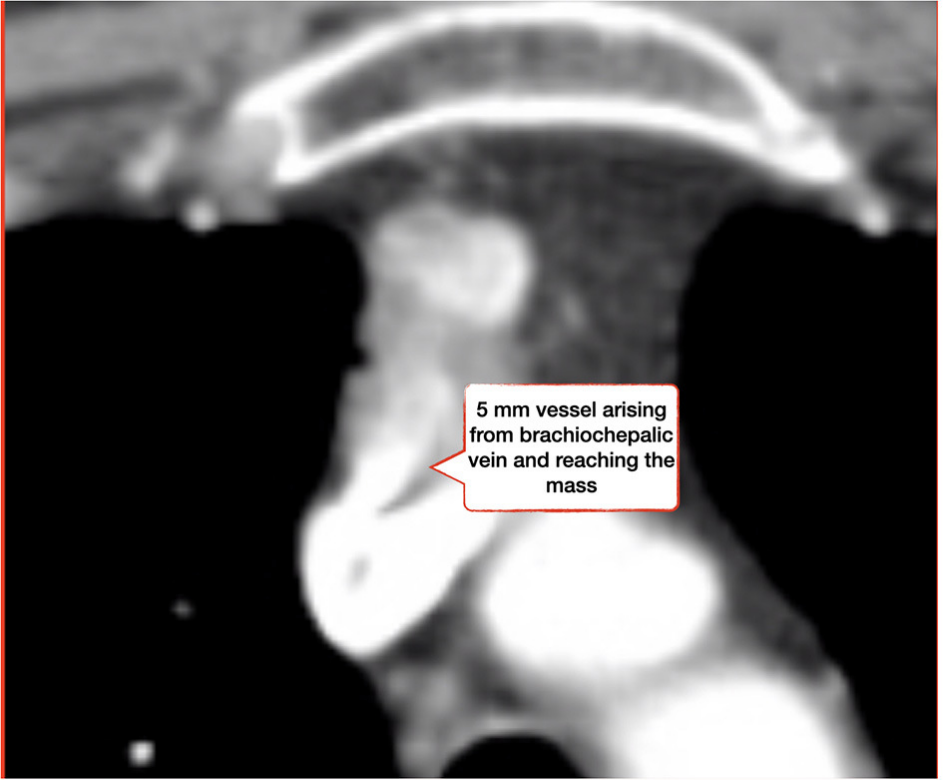
CT scan showing a 5 mm vessel arising from left brachiocephalic vein and reaching the mediastinal mass.

A 58 years-old man presented at our emergency room with fever, cough and left thoracic pain. His past medical history was unremarkable. Blood test showed WBC and C-reactive protein elevation. He had started antibiotics therapy and, given the persistence of symptoms, CT scan was performed showing lower left pneumonia. Incidentally, a 2.5 cm mass was found in the upper anterior mediastinum, with positive contrast enhancement and a 5 mm vessel arising from left brachio-cephalic vein (Figures 1, 2). Because of size and position fine needle aspiration nor anterior mediastinotomy were performed. Radiologic features were consistent with thymoma that usually appears as an ovoid or lobulated, smooth, well-marginated mass, projecting over the mediastinum typically protruding unilaterally, therefore patient underwent (1) neurologic evaluation that excluded myasthenia (2) PET/CT scan that showed the absence of any pathological uptake. However, thymoma was felt to remain a diagnostic possibility and patient was referred for radical surgery, with particular attention to abnormal vascularization. He underwent left thoracoscopy and, in spite of extreme attention, during pleural dissection between mediastinum and sternum, a sudden massive bleeding occurred (about 1,500 cc) requiring emergency conversion to median sternotomy. It was then clear that the bleeding came from tumor and not from brachiocephalic vein or its branches, as initially thought. After controlling the bleeding, we removed the mass but it was necessary to sacrifice the left laryngeal nerve.

Intra-operative emergency required significant blood transfusions and precautionary transfer in intensive care for 24 h. The patient eventually made a complete recovery. We only recorded transitory dysphonia as in case of laryngeal nerve paralysis. At specimen analysis, the cut surface seemed to have some fresh blood and the soft tissue was taupe with multifocal thrombosis in various stages of organization up to phlebolithes. Histopathologically, it was diagnosed a residual thymic tissue surrounded by lipomatous hyperplasia with venous malformation consisting essentially of a large number of dilated vessels with a single layer of endothelial cells with no signs of atypia or mitosis. Most of the channels contained red blood cells in the cavity. Margins were free. Definitive histology was a 7 cm fatty specimen enclosing a 2.5 cm in size mass diagnostic for residual thymus associated with venous vascular malformation (ISSVA classification, 2008).

## Discussion

### Surgical Assessment

We experienced a similar case 10 years ago (5). A 29-year-old man was diagnosed a huge tumor (about 10 cm in size) in the anterior mediastinum. At CT scan it was multilobulated, with flecks of calcification, in direct communication with the innominate vein and with contrast enhancement. CT guided percutaneous biopsy and anterior mediastinotomy were inadequate. Patient was therefore referred for radical surgery since angiosarcoma was thought to remain the more likely diagnosis. Surgery was complicated by sudden copious life-threatening intra-operative bleeding (1,400 mL) very demanding to be managed. Final diagnosis was: numerous thin walled blood vessels of variable size, closely packed within fibrous connective tissue, compatible with artero-venous malformation.

In our opinion, there are two challenges when dealing with vascular mediastinal mass. The first one is diagnostic and the second strictly related to surgical technique. Traditional non invasive imaging could help in differential diagnosis distinguishing vascular malformations from other masses as hemangioma, angiosarcoma, or even lymphatic malformations such as cystic hygromas or lymphangiomas (6) and differential diagnosis is sometimes very challenging even after repeated histologic examinations (diagnostic yield about 70%) (7). Therefore, it may happen to perform for surgery without tissue diagnosis, so that the surgeon is exposed to the risk of facing unexpected and challenging intraoperative situations without pre-defined strategy.

Moving to the second point, surgery for mediastinal vascular malformation may be particularly challenging due to adhesions on surrounding vital structures such as great vessels and major airways. Moreover, even tumor manipulation or traction could lead to its rupture with dramatic bleeding. That is why, surgery can be very demanding in some instances and preoperative embolization may be extremely useful to reduce blood flow within vascular component, decreasing technical challenges and lowering intraoperative bleeding risk (8).

### Radiologic Assessment

Although contrast enhanced CT examination plays a crucial role in the non-invasive diagnosis of mediastinal masses, their differential diagnosis is often based on pathological examination rather than on diagnostic imaging. Thymic cavernous hemangiomas belongs to the family of vascular malformations and, as such, typical CT-features such as multiple venous lakes, calcified phlebolithes, complex multiple venous channels, distant feeding veins and delayed enhancement are helpful to achieve a correct diagnosis (9). Particularly, calcified phlebolithes are a highly specific finding with a poor sensitivity, being observed in only 10% of thymic cavernous hemangioma cases (10). Although CT is a widely accessible diagnostic tool which offers a high spatial resolution and clearly demonstrates calcifications and phlebolithes, MRI is more suitable for characterizing the type of vascular malformation and, thanks to its excellent contrast resolution, is more accurate in depicting its extension and infiltration of the adjacent tissues (11). In addition, MRI is free from ionizing radiation. MRI patterns of vascular malformation include intermediate signal intensity on T1 weighted images and high T2-signal intensity with phlebolithes appearing as signal voids. Enhancement differs on the degree of vascularity, with arteriovenous malformations presenting more intense enhancement, while venous malformations show a typically slow but progressive enhancement (12).

Despite the diagnostic role of conventional digital subtraction angiography (DSA) has been reduced by the advent of new generation CT scans and MRI scans, it still plays a crucial role in the preoperative evaluation of such vascular malformations as it allows to map the arterial feeders, the draining veins and understand the hemodynamic characteristics. The therapeutic approach is chosen according to size, depth, and extent. As such, DSA can also be used as part of the therapeutic strategy of thymic vascular malformations, as vessel embolization allows to occlude arterial feeders and/or draining veins in order to facilitate surgery or to avoid it (13). The selection of the proper embolic agent is paramount. Common embolic agents employed to treat complex vascular malformations are liquid embolic system, both adhesive and non adhesive ones, as well as embolic particles and ethanol (14).

### Pathologic Assessment

According to the ISSVA (International Society for the Study of Vascular Anomalies) classification (2) vascular anomalies are distinct as vascular tumors or vascular malformations (VMs). Vascular tumors are characterized by endothelial hyperplasia, as Mulliken and Glowacki originally demonstrated (1). The term “hemangioma” should be restricted to their benign forms. Vascular malformations, on the other hand, are characterized by non-proliferative vascular channels, often ectatic, which are divided according to the involved vessels in arterial-venous, venous, capillary and lymphatic malformations, pure or variously combined.

Specifically, venous malformations are deviations in the development of the venous system caused by failures in different stages of embryogenesis. They are usually sporadic, but sometimes familiar. In familiar forms and in more than 50% of sporadic cases the malformation is linked to the activating mutation of the TEK gene; more rarely they are related to PIK3CA mutation (15, 16). They can present with pathological and clinical patterns of varying severity and are generally characterized by large, dilated vessels lined with flattened endothelium, occupied by low-flow blood which over time can organize itself into foci of local intravascular coagulation (LIC) and phlebolitis. Venous malformations have often been erroneously referred to as “cavernous hemangiomas” or “cavernous angiomas,” leading to confusion with true hemangioma. They have been found in any anatomical site, preferring limbs and craniofacial area. Superficial, cutaneous and mucous localizations are prevalent, but they are also observed with deep intramuscular, intraosseous or visceral localization as liver, gastrointestinal tract, and even thymus (17).

From literature review mediastinal venous malformations are uncommon. Although their origin in the mediastinum is variable, a small number showed thymic origin. Nowadays, only 10 cases of thymic so called “cavernous hemangioma” have been reported in English literature (9).

Histologically they must be mainly differentiated by thymic cystic lymphangioma, characterized by dilated lymphatic spaces consisting of endothelial cells comparable to cells lining normal lymphatics. Lymphatic spaces are usually filled with proteinaceous eosinophilic fluid and supporting stroma is composed of collagen and may contain lymphocytes and lymphoid aggregates. Their endothelial cells are positive for D2-40 immunohistochemical marker. Another differential diagnosis is with angiolipoma which is a rare benign tumor consisting of mature adipose tissue and blood vessels.

The strengths of this report is rarity of mediastinal cavernous hemangioma; a bias is the lack of images (such as coronal section, sagittal section images, dynamic contrast CT including unenhanced, early phase, and delayed phase, MRI; T1WI, T2WI, and diffusion-weighted images) that help readers to grasp the anatomy of the lesion in relation to adjacent tissues.

## Conclusions

Vascular malformations are benign structures, however some lesions may have invasive behavior, determining complications such as hemorrhage or asphyxia. Complete surgical resection is a radical treatment. Most of them have a complete capsule and are easy to dissect, so VATS can be used to remove lesions. Sometimes tumors are huge, or are closely stuck to large blood vessels or pericardium with an abundant blood supply making dissecting during thoracoscopy challenging. When the lesion is difficult to remove, the chest should be opened in time to ensure the safety of the operation. Percutaneous vascular embolization is used in the treatment of hemangioma, and, even though microinvasive surgery, is safe and effective. One of the most demanding topic is difficulty to obtain a pathological diagnosis before surgery.

In summary, when dealing with mediastinal masses, remember: (1) differential diagnosis between the more frequent solid antero-superior mediastinal tumors and vascular malformation should be always considered, especially in presence of abnormal vessels feeding into the mass (2) CT and MRI may not be conclusive and biopsy maneuvers are quite never definitive, sometime they could also be dangerous (3) preoperative DSA should not be spared in case of uncertain diagnosis (4) coil embolization should always be considered to reduce potentially fatal bleeding during surgery (5) histologic differentiation with other thymic neoplasms must be always considered (6) multidisciplinary approach is the key to guarantee the best and safest outcomes.

## Data Availability Statement

The raw data supporting the conclusions of this article will be made available by the authors, without undue reservation.

## Ethics Statement

Written informed consent was obtained from the patient for publication of this case report and any accompanying images. A copy of the written consent is available on request.

## Author Contributions

FR and MS: performed the operation, collected information of patient, revised the literature, and drafted the manuscript. LM: performed the pathologic examination. MC: performed radiological examination. AB, AG, and UC: revised the literature and revised the manuscript. All authors: contributed to the article and approved the submitted version.

## Conflict of Interest

The authors declare that the research was conducted in the absence of any commercial or financial relationships that could be construed as a potential conflict of interest.
